# Evaluation of *Muc1* Gene Expression at The Time of Implantation in Diabetic Rat Models Treated with Insulin, Metformin and Pioglitazone in The Normal Cycle and Ovulation Induction Cycle 

**DOI:** 10.22074/ijfs.2020.44409

**Published:** 2020-10-12

**Authors:** Ronak Zarei, Parvaneh Nikpour, Bahman Rashidi, Nahid Eskandari, Roshanak Aboutorabi

**Affiliations:** 1 Department of Anatomical Sciences, Faculty of Medicine, Isfahan University of Medical Sciences, Isfahan, Iran; 2 Department of Genetics and Molecular Biology, Faculty of Medicine, Isfahan University of Medical Sciences, Isfahan, Iran; 3 Child Growth and Development Research Center, Research Institute for Primordial Prevention of Non-communicable Disease, Isfahan University of Medical Sciences, Isfahan, Iran; 4 Department of Immunology, Faculty of Medicine, Isfahan University of Medical Sciences, Isfahan, Iran

**Keywords:** Diabetes Mellitus, Embryo Implantation, *Muc1*, Ovulation Induction

## Abstract

**Background:**

Mucin-1(Muc1) is one of the first molecules in the endometrium that confronts implanting embryos. There is insufficient knowledge about the impacts of diabetes and drugs developed for diabetes treatment on expres- sion of this molecule at the time of implantation. Therefore, this study aimed to investigate the impacts of diabetes and insulin, metformin and pioglitazone on Muc1 expression at the time of implantation.

**Materials and Methods:**

This experimental study was conducted on a total of 63 female Wistar rats divided into 9 groups. To induce type 1diabetes, streptozotocin (STZ) and for induction of type 2 diabetes, nicotinamide (NA) and STZ were injected intraperitoneally. For superovulation, human menopausal gonadotropin (HMG) and human chori- onic gonadotropin (HCG) were used. Insulin, metformin and pioglitazone were administered for two weeks. Finally, the endometrial expression of Muc1 was evaluated by quantitative real-time reverse transcription–polymerase chain reaction (RT-PCR).

**Results:**

*Muc1* expression was non-significantly increased in type 1 and type 2 diabetic groups compared to the con- trol group (P=0.61 and 0.13, respectively); also, it increased in insulin-treated type 1 diabetic group compared to the control group (P=0.0001). Its expression was increased in insulin-treated type 1 diabetic group compared to untreated diabetic group (P=0.001). The expression level of Muc1 was significantly reduced in superovulated and insulin-treated type 1 diabetic group compared to the insulin-treated type 1 diabetic group (P=0.001).

**Conclusion:**

One of the causes of fertility problems in diabetes, is changes in *Muc1* expression during implantation. On the other hand, the use of insulin in these patients can even lead to overexpression of this gene and worsen the condition. However, these changes can be partially mitigated by assisted reproductive technology (ART) such as su- perovulation. Also, treatment with metformin and pioglitazone can restore *Muc1* expression to near normal levels and has beneficial effects on implantation.

## Introduction

Diabetes mellitus is a metabolic disorder which is basically characterized by a chronic hyperglycemic condition. Type 1 diabetes predominately affects individuals of younger ages and type 2 diabetes as the most common type of diabetes, was previously thought to affect the ages of 40-60 years (1). The onset of type 2 diabetes occurs at younger ages (fertility age) today and it is predicted to occur at even younger ages in the future (2). Diabetes affects women in many ways and the association between diabetes and infertility was shown (3). Numerous studies observed that the incidence of infertility is higher in women with diabetes than in healthy women (4, 5).

Increased maternal blood glucose caused by diabetes can have detrimental effects on the expression of genes involved in the implantation process (6). However, the exact mechanism contributing to early pregnancy failure and recurrent spontaneous abortion in diabetes, remains largely unknown (7). Based on the emerging investigations, implantation failure is the main reason of about 75% of pregnancy losses (8). Embryo and uterus molecular crosstalk is the key factor for a successful pregnancy in the implantation process (9).

Muc1 as an anti-adhesion and antibacterial protein, is mainly expressed in the luminal and glandular endometrial epithelium at different stages of the menstrual cycle (10). Increment of *Muc1* expression before implantation leads to prevention of the embryo adhesion. Then, at the initiation of receptivity period of endometrium, *Muc1* is reduced and endometrium comes into contact with blastocyst. Therefore, timely inhibition of *Muc1* expression plays an important role in the uterine receptivity (11, 12). Actually *Muc1* hides the expression of cell adhesion molecules that are important for blastocyst attachment and plays an important role in regulating endometrial acceptance for blastocyst implantation (12). *Muc1* expression during implantation window in the endometrium of recurrent implantation failure women, is significantly lower than normal women (13). *Muc1* is an important factor in determining uterine receptivity and its endometrial expression is required for selection and implantation of the high-quality and active blastocysts. Significant decreases in Muc1 can impair endometrial embryo selection and lead to subfertility (11). On the other hand, increases in Muc1 in cell surface can inhibit cell-cell adhesion (14).Therefore, dysregulation of the mechanisms involved in the expression of *Muc1* at the time of implantation, may prevent implantation and establishment of early pregnancy.

Ovulation induction or superovulation in a controlled manner, is the most common method of assisted reproductive technology (ART). Various studies observed that infertile patients undergoing ART such as ovulation induction, experience molecular changes in their endothelium which can impair the expression of genes engaged in the embryonic implantation (15). Medications used to control diabetes include insulin for type 1 diabetes and oral medications such as metformin and pioglitazone and ultimately insulin for type 2 diabetes (16).

Studies demonstrated that *Muc1* expression in the endometrium is very important at the time of implantation, but there is insufficient knowledge about how this gene is expressed under diabetic conditions and the impacts of diabetes treatment and superovulation on the expression of this gene, need further assessments. Therefore, this study aimed to investigate the impacts insulin, metformin and pioglitazone as well as superovulation on the expression profile of *Muc1* during the implantation process, by using experimental rat diabetes model (type 1 and type 2 diabetes).

## Materials and Methods

This experimental study was done in female Wistar rats (6-8 weeks old; 200-250 g; obtained from Pasteur Institute, Iran). Animals were exposed to standard conditions, 12 hours light/dark cycle and 20-2°C, and they had free access to standard water and food. They were housed in the central animal laboratory of Isfahan University of Medical Sciences, Isfahan, Iran. All experimental processes were approved by the Institutional Animal Ethics Committee of Isfahan University of Medical Sciences (IR.MUI.REC.1396.3.366).

### Diabetes induction

To induce type 1 diabetes, streptozotocin (STZ, Sigma-Aldrich, Germany) was administered intraperitoneally at a dose of 60 mg/kg. For induction of type 2 diabetes, nicotinamide (NA, Sigma-Aldrich, Germany) was injected intraperitoneally at a dose of 200 mg/kg and after 15 minutes, STZ 60 mg/kg was given (17). To confirm diabetes induction, fasting blood sugar (FBS) was determined 3 days after the injection(s) by a glucometer (HemoCue Glucose 201+, Sweden) in samples collected from the dorsal vein of rats. In this study, in case of an FBS> 250 mg/dl, diabetes induction was confirmed (18).

### Ovulation induction

Human menopausal gonadotropin (HMG; N. V. Organon, The Netherlands) and human chorionic gonadotropin (HCG, N. V. Organon, The Netherlands) was used for ovulation induction. Three days before mating, first, HMG was injected intraperitoneally at 7.5 I.U. and 48 hours later, HCG was injected at 7.5 I.U. in the same manner (19).

### Study design and sample collection

Diabetic and normal rats were randomly divided into 9 groups: control (healthy animals that received no treatments), type 1 diabetic rats induced by STZ that received no treatments, insulin-treated type 1 diabetic rats, superovulated rats induced by HMG/HCG, superovulated type 1 diabetic rats, superovulated and insulin-treated type 1 diabetic rats, type 2 diabetic rats induced by NA-STZ that received no treatments, 20 mg/kg/day pioglitazone (Sobhan, Iran)-treated diabetic rats (20), and 100 mg/kg/day metformin (Sobhan, Iran) -treated diabetic rats (21). There were 7 rats in each group and animals were kept in diabetic conditions for 4 weeks (for more than one sex cycle), and administered with drugs for 4 weeks. During all diabetic conditions and treatments, FBS levels were monitored by a glucometer (HemoCue Glucose 201+, Sweden) and glucose reagent strips (ACCU-CHEK Active, Germany), every 4 days.

Four days earlier than the end of the treatment period, two female rats of each group were mated with a male rat and vaginal plug was checked in the following morning. The day when the vaginal plugs were observed or vaginal smears showed spermatozoa, was considered the first day of pregnancy. Rats were fasted overnight during the 3rd night and anesthetized through intraperitoneal injection of ketamine hydrochloride (50 mg/kg; ROTEXMEDICA, Germany) and xylazine hydrochloride (7 mg/kg; Daroupakhsh, Iran) on the following day; then, they were sacrificed under sterile conditions on the 4^th^ day of gravidity (the day of implantation). Uterine horns were surgically separated and snap-frozen in liquid nitrogen and stored at -80°C for further investigations.

### Total ribonucleic acid isolation and complementary DNA synthesis

Total ribonucleic acid (RNA) was extracted from endometrial tissue by RNX-plus (Sinaclon, AryoGen Biopharma Complex, Iran) according to the manufacturer's protocol. Purity was defined by 1% agarose gel electrophoresis. The total RNA concentration was measured using a Nanodrop device (Nanolytic, Germany) at a density of 260 nm. DNase Ι treatment was accomplished in order to remove genomic DNA in the RNA samples by DNase Ι set (Fermentas, Lithuania). Complementary DNA (cDNA) synthesis was conducted using 1 μg of total RNA, by means of PrimeScriptTM RT reagent Kit (TaKaRa, Kusatsu, Japan) as reported in the protocol (22).

### Quantitative real-time reverse transcription polymerase chain reaction

The relative expression level of *Muc1* gene was measured by real-time reverse transcription polymerase chain reaction (RT-PCR) in comparison with β-actin as a reference gene. The primers were planned using GeneRunner software (Version 4.0; Hastings Software Inc., Hastings, US) and the specificity of each primer was tested by BLAST (http://blast. ncbi.nlm.nih.gov/Blast.cgi). The list of primers is presented in Table 1 (23).

RT-PCR was performed by Applied BiosystemsStepOne- Plus™ instrument using RealQ Plus ×2 Master Mix, green (high ROX) (AMPLIQON, Denmark) (24). Standard cycling protocol was utilized to perform RT-PCR, as follows: denaturation at 95°C for 10 minutes, denaturation at 95°C for 15 seconds, annealing at the specific temperature for each gene (Table 1) for 60 seconds, and finally, an extension was done for 15 seconds at 72°C for 40 cycles. Gene expression determination was carry out using the 2^-ΔΔCT^ method (25).

### Statistical analysis

All statistical analyses were done by using SPSS software, version 20.0 (SPSS Inc., US). To analyze the normality of the data, Kolmogorov-Smirnov test was applied. RT- PCR was repeated three times and the final results are shown as means ± standard error of the mean. One-way Analysis of Variance (ANOVA) with post hoc LSD multiple comparisons were accomplished to recognize statistical significance. Statistical significance was set at P<0.05.

**Table 1:** PCR primer sequences


Primers	Sequence	Tm (ºC)	Annealing temperature (ºC)	Amplicon size (bp)

*βactin*	F: 5´-GCCTTCCTTCCTGGGTATG-3´ R: 5´-AGGAGCCAGGGCAGTAATC-3´	63.4 63	60	178
*Muc1*	F: 5´- ATCAAGTTCAGGTCAGGCTC -3´R: 5´- AGAGGAAGGGAACTGCATC-3´	60.1 59.9	57	171


## Results

### *Muc1* gene expression in type 1 diabetic and superovulated groups compared to the control group

Relative expression of *Muc1* was increased in type 1 diabetic and insulin-treated type 1 diabetic groups compared with the control group; however, statistically significant differences were only found for insulin-treated type 1 diabetic group (P=0.0001, 0.61; respectively). The other groups (superovulated, superovulated type 1 diabetic and superovulated and insulin-treated type 1 diabetic groups) did not show a significant difference when compared with the control group (P=0.51, 0.78, 0.95, respectively).

**Fig.1:**
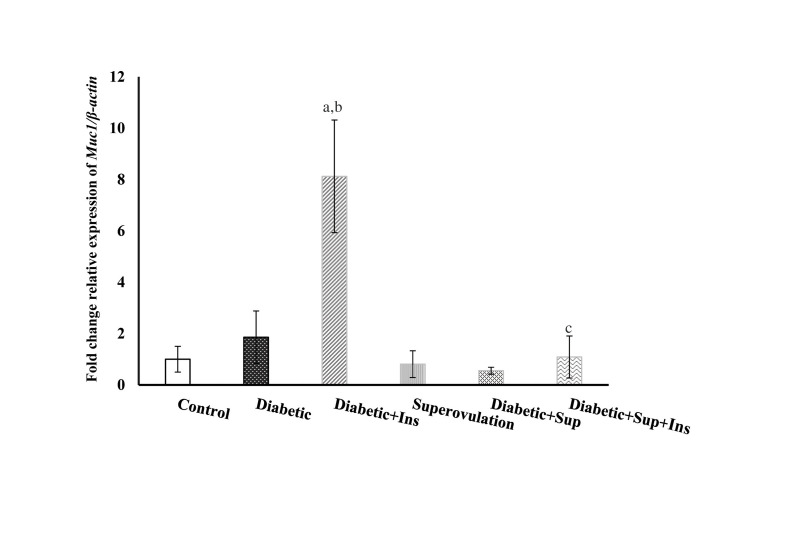
Relative expression of *Muc1* in the endometrium of type 1 diabetic rats at the time of implantation. The relative expression of *Muc1* was normalized against β-actin using 2^-∆∆CT^ method. All values are presented as mean ± SEM. A P<0.05 was considered statistically significant. SPSS software was used to analyze the data. Lowercase letters indicate a statistical significance as follows: a: Compared to control, b: Untreated diabetic, c; Insulin-treated diabetic groups. Sup; Superovulation, and Ins; Insulin.

*Muc1* expression in insulin-treated type 1 diabetic group increased compared to the untreated diabetic group (P=0.001). In superovulated and insulin-treated type 1 diabetic groups, relative expression of *Muc1* gene was significantly reduced compared to the insulin-treated type 1 diabetic group (P=0.001, Fig .1).

### *Muc1* gene expression in type 2 diabetic groups compared to the control group

Type 2 diabetic group showed increment (though not significantly) of the expression of *Muc1* compared to the control group (P=0.13). Relative expression level of *Muc1* was not significantly different between metformin-treated and pioglitazone-treated type 2 diabetic groups, and the control group (P=0.94, 0.75; respectively).

**Fig.2:**
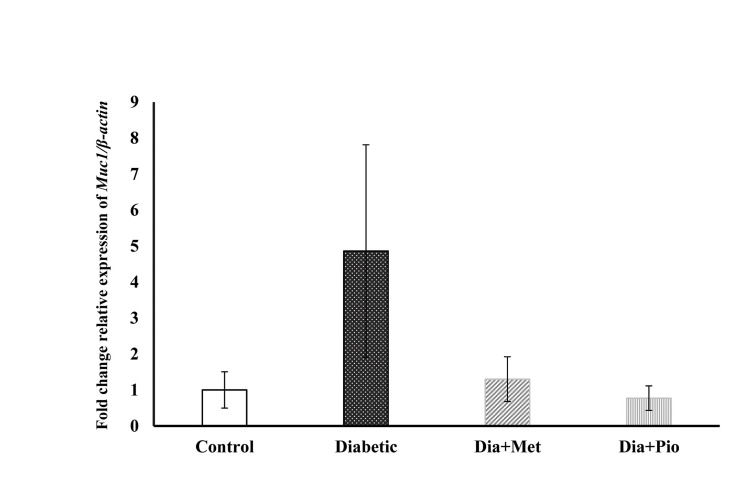
Relative expression of *Muc1* in the endometrium of type 2 diabetic rats at the time of implantation. The relative expression of *Muc1* was normalized against β-actin using 2-∆∆CT method. All values are presented as mean ± SEM. A P< 0.05 was considered statistically significant. SPSS software was used to analyze the data. Dia; Diabetic, Met; Metformin, and Pio; Pioglitazone.

*Muc1* expression was non-significantly reduced in type 2 diabetic groups treated with metformin and pioglitazone compared to untreated type 2 diabetic group (P=0.11, 0.07; respectively, Fig .2).

## Discussion

According to the results of the present study, induction of type 1 and type 2 diabetes increased the expression of *Muc1* in rats’ endometrium at the time of implantation. Both metformin and pioglitazone had positive effects on restoration of *Muc1* expression to normal levels but insulin caused overexpression of *Muc1*. However, ovulation induction partially moderated the effect of insulin and *Muc1* expression level became closer to normal.

The results of the current study showed that induction of type 1 and type 2 diabetes increased *Muc1* gene expression in rats’ endometrium. *In vitro* studies indicated that Muc1 is reduced in humans and mice specifically in the area where the blastocyst implants in the uterus. It is hypothesized that low level of Muc1 in the blastocyst implantation area during implantation window, is an important factor for successful embryo-endometrial interaction. High expression of Muc1 may damage cell-cell and cell-matrix adhesion, probably leading to implantation failure (26). Aktug et al. (14)study showed that induction of diabetes affects cleaved junctions, cell adhesion molecules and related proteins. They fertilized the oocytes isolated from the healthy and diabetic rats and found that Muc1 expression was increased in a group of blastocysts in which, oocytes were separated from diabetic rats. Albaghdadi et al. (27). also observed the overexpression of *Muc1* in the uterus of diabetic mice at the time of implantation. In fact, the present study also confirmed these observations and showed that diabetes can increase *Muc1* expression during implantation which can lead to implantation failure.

The present study showed that treatment with insulin in type 1 diabetic rats, increased *Muc1* expression to a higher level compared to untreated diabetic rats, which may result in prevention of blastocyst contact with uterine epithelium and prevention of implantation. In Seregni et al. (28) study, insulin was found to increase the level of Muc1 expression in the blood of patients with breast cancer. The present study, consistent with these results, indicated that Muc1 overexpression caused by treatment with insulin during implantation can lead to implantation failure.

In the present study, treatment with either metformin or pioglitazone was effective in reducing *Muc1* expression levels in diabetic rats treated with metformin or pioglitazone compared with untreated diabetic rats. No studies were found on the effect of metformin or pioglitazone on *Muc1* expression at the time of implantation, under diabetic conditions. However, there is some evidence that metformin reduces MUC1 protein in women with breast cancer (29).

Furthermore, the results of the present study showed that ovulation induction in all induced groups including healthy, diabetic and insulin-treated diabetic rats, reduced Muc1 expression, although it was not significantly different from the control group. However, comparing insulin-treated diabetic rats with superovulated insulin-treated diabetic rats may be important since ovulation induction may possibly modulate insulin-induced increment of *Muc1* expression. Inyawilert et al. found that ovulation induction attenuated *Muc1* mRNA expression in the rat uterus on day 3.5 of the estrous cycle (30). Contrary to the present study, Wang et al. found that *Muc1* expression was artificially increased in ovine following ovarian stimulation, that may be due to difference in method of superovulation and the type of drug used to induce ovulation (31). Nonetheless, further studies are required to determine the effects of ovulation induction on *Muc1* expression and implantation.

According to the results of the current study, it can be concluded that type 1 and type 2 diabetes alter the expression of *Muc1* gene in the rat uterus at the time of implantation. Because of the importance of proper expression of *Muc1*, its aberrant expression may affect uterine receptivity and lead to implantation failure and subsequent infertility.

Both anti-diabetic drugs metformin and pioglitazone, had positive effects on restoration of *Muc1* expression to its normal levels. Inevitable treatment with insulin in type 1 diabetes caused overexpression of *Muc1*; however, ovulation induction partially moderated such effects and restored *Muc1* levels closer to normal values. However, ovulation induction alone may have adverse effects on the expression of this molecule.

There were limitations in this study, and the examined effects should be assessed in a larger number of rats in future works; also, follow-up of animal pregnancy to investigate the effects of medications on pregnancy outcomes was not possible in the current study.

## Conclusion

The use of insulin by diabetic patients, can even lead to overexpression of *Muc1* and worsen the condition. However, these changes can be partially mitigated by ART such as superovulation. Also, treatment with metformin and pioglitazone can restore *Muc1* expression closer to normal levels and have beneficial effects on implantation. Therefore, it can be said that diabetes can alter *Muc1* gene expression which can disrupt the implantation process and consequently induce infertility. However, treatment with metformin and pioglitazone as well as ovulation induction, can be helpful.
